# Changes in Lipid Profiles with the Progression of Pregnancy in Black Women

**DOI:** 10.3390/jcm13102795

**Published:** 2024-05-09

**Authors:** Nadia Saadat, Fernando Aguate, Alexandra L. Nowak, Suzanne Hyer, Anna B. Lin, Hannah Decot, Hannah Koch, Deborah S. Walker, Todd Lydic, Vasantha Padmanabhan, Gustavo de los Campos, Dawn Misra, Carmen Giurgescu

**Affiliations:** 1 Department of Pediatrics, University of Michigan, Ann Arbor, MI 48019, USA; vasantha@umich.edu; 2 Department of Epidemiology and Biostatistics, Michigan State University, East Lansing, MI 48824, USA; 3 School of Nursing, Loyola University, Maywood, IL 60153, USA; anowak8@luc.edu; 4 College of Nursing, University of Central Florida, Orlando, FL 32826, USA; 5 Molecular Metabolism and Disease Mass Spectrometry Core, Michigan State University, East Lansing, MI 48824, USA; 6 College of Nursing, Wayne State University, Detroit, MI 48202, USA

**Keywords:** African American women, pregnancy, metabolism, lipid profiles, lipid biomarkers

## Abstract

**Background/Objectives**: Lipid metabolism plays an important role in maternal health and fetal development. There is a gap in the knowledge of how lipid metabolism changes during pregnancy for Black women who are at a higher risk of adverse outcomes. We hypothesized that the comprehensive lipidome profiles would show variation across pregnancy indicative of requirements during gestation and fetal development. **Methods**: Black women were recruited at prenatal clinics. Plasma samples were collected at 8–18 weeks (T_1_), 22–29 weeks (T_2_), and 30–36 weeks (T_3_) of pregnancy. Samples from 64 women who had term births (≥37 weeks gestation) were subjected to “shotgun” Orbitrap mass spectrometry. Mixed-effects models were used to quantify systematic changes and dimensionality reduction models were used to visualize patterns and identify reliable lipid signatures. **Results**: Total lipids and major lipid classes showed significant increases with the progression of pregnancy. Phospholipids and glycerolipids exhibited a gradual increase from T_1_ to T_2_ to T_3_, while sphingolipids and total sterol lipids displayed a more pronounced increase from T_2_ to T_3_. Acylcarnitines, hydroxy acylcarnitines, and Lyso phospholipid levels significantly decreased from T_1_ to T_3_. A deviation was that non-esterified fatty acids decreased from T_1_ to T_2_ and increased again from T_2_ to T_3_, suggestive of a potential role for these lipids during the later stages of pregnancy. The fatty acids showing this trend included key fatty acids—non-esterified Linoleic acid, Arachidonic acid, Alpha-linolenic acid, Eicosapentaenoic acid, Docosapentaenoic acid, and Docosahexaenoic acid. **Conclusions**: Mapping lipid patterns and identifying lipid signatures would help develop intervention strategies to reduce perinatal health disparities among pregnant Black women.

## 1. Introduction

Pregnancy is a complex and dynamic metabolic state that involves significant changes in lipid metabolism to support overall maternal health and the growth of the fetus [1,2]. The metabolic state during pregnancy is constantly changing to meet the requirements of pregnancy progression and fetal growth [1,2]. The maternal body increases stores of fats and nutrients in the early stages of pregnancy [3]. During the early part of the pregnancy, plasma cholesterol levels are 1.5 times higher than pre-pregnancy levels, and triglyceride levels double, ensuring fetal substrate availability [3]. Stored lipids enter a catabolic state, providing nutrients for fetal growth and development in the later part of the pregnancy [1]. Considering that changes in lipid metabolism are critical for normal pregnancy adaptations and physiological responses including fat accumulation, mobilization, and transport, a better understanding of the changes in the lipidome throughout pregnancy would be beneficial in identifying biomarkers for early detection of pregnancy-related complications. This is especially important in African American/Black women, who experience a higher risk of adverse pregnancy outcomes, including preterm birth, low birth weight, and gestational diabetes, compared to other racial/ethnic groups [4,5]. Despite the critical role of lipids in pregnancy, the routine monitoring of lipid profiles is not currently in practice in prenatal care. Therefore, there is a need to better understand the changes in lipid metabolism that occur during pregnancy in Black women to identify potential lipid biomarkers for early detection of pregnancy-related complications.

Lipids have many roles in metabolism including serving as an energy reservoir and they are also major components of cell and plasma membranes [6]. Other vital functions of lipids include signal transduction, chemical messaging, and interaction and regulation of proteins [6]. Lipid levels and patterns have been associated with adverse pregnancy outcomes (e.g., preterm birth—<37 completed weeks gestation); small for gestational age (SGA, <10th percentile for GA); and large for gestational age (LGA, >90th percentile for GA) infants) [7,8,9]. Maternal lipid levels are reported to be linked to pregnancy outcomes including birth weight [10]. Adank et al. (2020) reported positive associations between maternal triglyceride and remnant cholesterol (cholesterol not related to high density lipoprotein cholesterol, HDL-C, and low density lipoprotein cholesterol, LDL-C) levels in early pregnancy with increased fetal head and abdominal circumference, growth rate, as well as the risk for LGA babies [8]. The researchers reported that these associations were independent of maternal body mass index (BMI) but related to maternal blood glucose levels [8]. In a secondary analysis of 1337 women, Kim et al. (2021) found that a higher ratio of cholesterol/lathosterol (suggesting increased cholesterol metabolism) in the first trimester of pregnancy was associated with SGA neonates. A longitudinal study of women recruited early in pregnancy (6–10 weeks gestation) found a significant inverse association between birth weight adjusted for gestational age and HDL-C at all timepoints in overweight/obese women but not in normal weight women. On the contrary, triglyceride levels at 10–14 and 22–26 weeks gestation were positively associated with adjusted birth weight for normal-weight women but not for overweight/obese women [11]. A recent study of pregnant women in Puerto Rico found associations of several phospholipids and LGA neonates [12]. The researchers also reported an association of plasmenyl-phosphatidylethanolamines and mono- and polyunsaturated free fatty acids with increased risk for preterm birth [12]. These findings suggest that subtle changes in lipid metabolism may affect pregnancy outcomes, highlighting the need for comprehensive lipid screening prior to and throughout pregnancy to identify any aberrant changes in lipid metabolism.

Black women have higher rates of diseases related to dyslipidemia including cardiovascular diseases, hypertension, obesity, and other metabolic disorders compared to non-Hispanic White women [13,14]. Black women are also more likely to develop coronary heart disease (CHD) and have higher CHD mortality compared to non-Hispanic White women [15]. In a case–control study with 29 Black women, the upregulation of specific triglyceride species and downregulation of specific phosphatidylcholine species were reported in the women with preterm birth compared to those with term births [16]. These findings provide evidence that tracking changes in lipidome profiles during pregnancy could be an important tool to identify risk for adverse pregnancy outcomes especially in Black women that are not well studied, thus emphasizing a strong need to establish the baseline lipid metabolism profile during the progression of pregnancy.

To address this gap in the literature in Black women and better understand changes in lipid metabolism as pregnancy progresses, we capitalized on an untargeted lipidome analysis approach to develop a comprehensive untargeted lipidome profile throughout pregnancy and identify lipid metabolism markers. This is one of the few studies to report changes in comprehensive untargeted lipidome profiles during pregnancy in Black women in the United States. We hypothesized that total plasma lipid levels will increase with the normal progression of pregnancy representing the increased demand of lipid metabolites and that lipid classes with specific functions would show variation across pregnancy reflecting their specific roles during gestation and fetal development. While lipid panel profiling captures only a few classes of lipids (e.g., low-density lipoproteins and triglycerides) our comprehensive lipidome screening approach simultaneously investigates all lipids. This state-of-the-art approach allows for the detailed identification of patterns and biomarkers of lipid metabolism modifications, thus giving a snapshot of the entire set of lipids and related metabolism at three timepoints [17]. We classified lipids based on the LIPID MAPS, the International Lipid Classification, and the National Nomenclature Committee [6].

## 2. Materials and Methods

### 2.1. Study Design and Sample

The pregnant Black women were recruited for this project at prenatal clinics in the Midwest (Detroit, MI, USA and Columbus, OH, USA) as a part of an ongoing NIH-funded study—the Biosocial Impact on Black Birth (BIBB) study—which used a prospective longitudinal design. Pregnant Black women were enrolled after 8 weeks of pregnancy up to the early 2nd trimester of pregnancy at a prenatal visit. The participants signed an informed consent form; completed a survey about sociodemographic characteristics and social and behavioral factors during and before pregnancy; and had blood samples collected. Questionnaire data and blood samples were collected at three timepoints during pregnancy: 8–18 weeks (T_1_), 22–29 weeks (T_2_), and 30–36 weeks (T_3_).

#### 2.1.1. Inclusion and Exclusion Criteria

Women were enrolled in the BIBB study if they (a) self-identified as African American /non-Hispanic Black; (b) were at least 18 years of age; (c) had a singleton pregnancy; (d) were of any parity; and (e) were English speaking. Women were excluded if they had a multiple gestation pregnancy (e.g., twins). Likewise, we did not include women above 45 years of age due to metabolic changes associated with this age group which might affect the metabolism of these women [18,19]. For this project, data from 64 Black women (60 women had samples at all 3 timepoints and 4 had samples at 2 timepoints, T_1_ = 64, T_2_ = 63, and T_3_ = 61) who had term births (completed 37 weeks of pregnancy) and plasma samples collected at the prenatal visits were used to investigate the changes in lipidome profiles with the progression of pregnancy.

#### 2.1.2. Self-Reported Questionnaire

Maternal sociodemographic characteristics were collected by self-reported questionnaires (e.g., maternal age, level of education, and annual household income). Participants completed a questionnaire on an electronic tablet or a link sent to them at T_1_ (8–18 weeks). At T_2_ (22–29 weeks) and T_3_ (30–36), participants completed follow-up questionnaires. Term births were confirmed from the birth records.

#### 2.1.3. Plasma Samples

Venous blood was drawn into a sterile 6 mL EDTA tube through antecubital venipuncture (within 30 s) by the medical assistant at the prenatal visits and kept on ice/refrigerated before processing. Within 2 h of collection, blood samples were centrifuged at 1600× *g* for 15 min at 4 °C to collect plasma and plasma samples were aliquoted (~0.5 cc/aliquot). The samples were stored −20 °C at the clinic and transferred to −80 °C at the end of the day, where the samples were stored at −80 °C until lipidome analysis. The same protocol was followed at the T_2_ and T_3_ timepoints. Two plasma aliquots per participant for each timepoint were shipped (on dry ice) to Michigan State University laboratory for untargeted lipidome analysis.

### 2.2. Lipidome Analysis

#### 2.2.1. Sample Extraction for Lipidome Analysis

Plasma samples were thawed on ice and 25 microliter aliquots were spiked with 10 microliters of an internal standard and calibration mixture consisting of 50 μM each of di-myristoyl phospholipids phosphatidylglycerol (PG), phosphatidylethanolamine (PE), phosphatidylserine (PS), phosphatidic acid (PA), phosphatidylcholine (PC), 50 μM sphingomyelin SM (30:1), and 25 μM triacylglycerol TG (14:1/14:1/14:1). Three hundred microliters of −20 °C chilled methanol containing 0.01% BHT (butylated hydroxytoluene) was added to each sample followed by the addition of 1 mL of methyl tert-butyl ether (MTBE). The samples were then vortexed for 60 min at room temperature. Then, 250 microliters of water was added, and the samples were vortexed for an additional 15 min and then centrifuged for 15 min. The supernatants were collected in new test tubes and precipitated proteins were re-extracted as above. Extracts were dried overnight in a speedvac and resuspended in 250 microliters of isopropanol containing 0.01% BHT [20].

#### 2.2.2. Lipidome Profiles

Lipidome profiles were determined by “shotgun” Orbitrap high resolution/accurate mass spectrometry. Immediately prior to analysis, aliquots of each lipid extract were diluted 5-fold into isopropanol:methanol (2:1, *v*:*v*) containing 20 mM ammonium formate. Full-scan MS spectra at 100,000 resolution (defined at *m*/*z* 400) were collected on a Thermo Scientific LTQ-Orbitrap Velos mass spectrometer in both positive and negative ionization modes. Scans were collected from *m*/*z* 200 to *m*/*z* 1200. For each analysis, 10 µL of sample was directly introduced by flow injection [21] at 10 µL/min using an electrospray ionization source equipped with a fused silica ESI needle to minimize the intra-source accumulation of triglycerides. A Shimadzu Prominence HPLC autosampler with a thermostat served as the sample delivery unit. The sample and injection solvent were 2:1 (*v*:*v*) isopropanol: methanol containing 20 mM ammonium formate. The spray voltage was 4.5 kV, the ion transfer tube temperature was 275 °C, the S-lens value was 50 percent, and the Orbitrap fill time was 100 ms. The autosampler was set to 4 °C. After two minutes of MS signal averaging, the LC tubing, autosampler, and ESI source were flushed with 1 mL of isopropanol, prior to injection of the next sample. Samples were analyzed in a random order, interspersed by solvent blank injections, extraction blank injections, and pooled QC samples derived from all study samples. Following MS data acquisition, offline mass recalibration was performed with the “Recalibrate Offline” tool in Thermo Xcalibur software (https://www.thermofisher.com/us/en/home/industrial/mass-spectrometry/liquid-chromatography-mass-spectrometry-lc-ms/lc-ms-software/lc-ms-data-acquisition-software/xcalibur-data-acquisition-interpretation-software.html) according to the vendor’s instructions, using the theoretical computed masses for the internal calibration standards and several common endogenous mammalian lipid species. MS/MS confirmation and structural analysis of lipid species identified by database searching were performed using higher-energy collisional dissociation (HCD) MS/MS at 60,000 resolution and a normalized collision energy of 25 for the positive ion mode and 60 for the negative ion mode. MS/MS scans were triggered by inclusion lists generated separately for positive and negative ionization modes. 

#### 2.2.3. Lipid Peak Finding, Identification, and Quantitation

Lipids were identified using the Lipid Mass Spectrum Analysis (LIMSA) v.1.0 software linear fit algorithm, in conjunction with an in-house database of hypothetical lipid compounds, for automated peak finding and correction of ^13^C isotope effects as previously described [22]. The peak areas of found peaks were quantified by normalization against an internal standard of a similar lipid class. The top ~300 most abundant peaks in both positive and negative ionization mode were then selected for MS/MS inclusion lists and imported into Xcalibur software for structural analysis on the pooled QC sample as described above. For this untargeted analysis, no attempt was made to correct for differences in lipid species ionization due to the length or degree of unsaturation of the esterified fatty acids. Therefore, lipid abundance values are inherently estimates rather than true “absolute” values.

### 2.3. Statistical Analysis

The data were maintained on a dedicated password-protected server with encrypted files.

To visualize overall trends in lipidome profiles, dimensionality reduction modeling was performed. Multivariate models were generated using SIMCA 17 (Sartorius Stedim Data Analytics AB). The data were scaled by unit variance scaling. For an overview of the data, unsupervised models (principal component analysis—PCA) were developed. Supervised models (partial least squares discriminant analysis—PLS DA and orthogonal partial least squares discriminant analysis—OPLS DA) were used to investigate differences in timepoint groupings and to identify the potential lipid signatures of each timepoint.

We used mixed-effects models to estimate the within-subject correlation of lipid abundances and to quantify systematic changes in lipid abundances over time. The mixed-effects model included the time-point means ($\beta_{j};\;j=1,2,3)$ plus the random effect of the subject ($u_{i};i=1,\ldots ,n$). Therefore, the longitudinal model for the abundance of a lipid had the following form.

$$M1:\;y_{ij}=\mu_{j}+u_{i}+\varepsilon_{ij}.$$

The time-point means were treated as fixed effects; the random effects of the subject and the error terms were assumed to be independent, both normally distributed, $u_{i}\sim N\left(0,\sigma_{u}^{2}\right)$ and $\varepsilon_{ij}\sim N\left(0,\sigma_{\varepsilon }^{2}\right)$, respectively.

The within-subject correlation of repeated measures is $Cor\left(y_{ij},{y_{ij}}^{\prime }\right)=\frac{\sigma_{u}^{2}}{\sigma_{u}^{2}+\sigma_{\varepsilon }^{2}}$.

To test for time-point effects, we fitted the model with a common mean across time-points,

$$M0:\;y_{ij}=\mu +u_{i}+\varepsilon_{ij}$$

We tested for significant changes in lipid classes across time-points by performing a (2-df) likelihood ratio test between models M1 and M0.

We applied the above approach to 18 lipid classes. For each of these analyses, we present estimates of intra-class correlation and *p*-values for the effect of time on abundances. Within each level, significance was determined using a family-wise error rate of 0.05, adjusted to account for multiple testing using Bonferroni’s method. All these analyses were performed using R (R Development Core Team 2019), the mixed-effects models were fitted using the lme4 package [23], and plots were produced using ggplot2 [24].

### 2.4. Maternal Characteristics

Women in this study had a mean age of 27.17 ± 5.68 years. More than half of the participants (51.6%) had an annual household income < USD 20,000 and half of them were working (50.0%); 57.8% of participants were never married and 4.7% were separated; and 87.5% of them had a high school or higher level of education (Table 1).

## 3. Results

**Figure 1:**
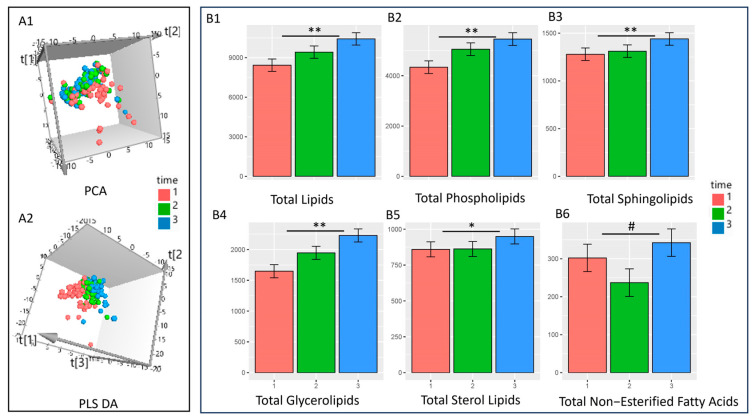
(**A**) The 3D dimensionality reduction models showing overall differences in lipidome profiles with the progression of pregnancy. (**A1**)—unsupervised principal component score plot. (**A2**)—partial least square discriminant analysis score plot. One score represents one sample. Timepoints T_1_ (red), T_2_ (green), and T_3_ (blue). (**B**)—Bar graphs showing significant differences in the levels of lipids at three timepoints using mixed-effects models: (**B1**)—An overall increase in total lipid levels was observed with the progression of pregnancy (FDR-adj *p* = 8.05 × 10^−9^. (**B2**)—Total phospholipid levels increased (FDR-adj *p* = 4.53 × 10^−9^). (**B3**)—Total Sphingolipids also increased with the progression of pregnancy (FDR-adj *p* = 0.000714). (**B4**)—Total glycerolipids increased with the progression of pregnancy (FDR-adj *p* = 4.38 × 10^−10^). (**B5**)—Total sterol lipid levels increased at timepoint 3 (FDR-adj *p* = 0.0326). (**B6**)—Total non-esterified fatty acids showed a trend of decrease at T2 and increase at T3 timepoints with the progression of pregnancy (FDR-adj *p* = 0.0742) (* *p* < 0.05, ** *p* < 0.01, # *p* < 0.1).

In the overview analysis, the unsupervised dimensionality reduction principal component analysis (PCA, good model with good fit R2 = 0.8 and Q2 = 0.6) score plot showed a separation of T_1_ from T_2_ and T_3_; however, there was no clear separation observed between T_2_ and T_3_ in Figure 1(A1). The supervised partial least square discriminant analysis (PLS DA) model showed grouping and separation of all three groups, but the predictive ability of the model was lower compared to the unsupervised model (R2 = 0.6 and Q2 = 0.2) Figure 1(A2).

Total lipids and some of the major lipid classes showed a significant increase in the mixed-effects models (Figure 1B) with the progression of pregnancy. However, differing patterns were observed with lipid classes, where some lipid classes showed no significant difference across timepoints, some lipid classes showed a significant decrease in abundance during pregnancy, and others showed non-linear patterns over time, with a reduction during the first half of the pregnancy and an increase thereafter.

The lipid classes which showed a gradual significant increase from T_1_ to T_2_ and then T_2_ to T_3_ (Table 2) included PC, PE, very long-chain (≥C24, VLC) PE, PG, phosphatidylinositol (PI), cardiolipin (CL), ceramide (Cer), hexosylceramide (HexCer), lacotsylceramide (LacCer), SM, sulfatide (ST), diacylglydiceride (DG), TG, free cholesterol, esterified MUFA and PUFA, esterified Linoleic acid, total diradyl phospholipids, total choline-containing lipids, total amine phospholipids (PE and PS), and total glycolipids (PI, HexCer, LacCer, and ST). The lipid classes which showed a gradual decrease from T_1_ to T_2_ and then from T_2_ to T_3_ (Table 3) included Lyso PC, Lyso PE, Free d16:1, Free d18:0, Free d18:1, Free d18:2, Free d20:0, Free d20:1, d16:0-1-PO4, MG, VLC Cholesterol Esters, Acylcarnitines, Hydroxy acylcarnitines, and total lyso phospholipids. The lipids which showed a reduction in levels from T_1_ to T_2_ and then an increase in levels from T_2_ to T_3_ (Table 4) included polyunsaturated NEFA, non-esterified Linoleic acid (18:2n6), non-esterified Arachidonic acid (20:4n6), non-esterified ALA (18:3n3), non-esterified EPA (20:5n3), non-esterified DPA (22:5n3), and non-esterified DHA (22:6n3).

### 3.1. Total Lipid Levels and Major Classes of Lipids Increase with Progression of Pregnancy

Total lipid levels exhibited a significant increase from T_1_ to T_2_ and T_2_ to T_3_ (Figure 1B). Our results show a clear trend of increased total lipid levels with the progression of pregnancy (R^2^ = 0.78, FDR-adjusted *p*-value = 8.05 × 10^−9^, Figure 1). Further analysis revealed that plasma levels of specific lipid classes also significantly increased with pregnancy progression. Phospholipids (R^2^ = 0.773, FDR-adjusted *p*-value = 4.53 × 10^−9^) and glycerolipids (R^2^ = 0.707, FDR-adjusted *p*-value = 4.38 × 10^−10^) exhibited a gradual increase throughout the pregnancy, while sphingolipids (R^2^ = 0.780, FDR-adjusted *p*-value = 7.14 × 10^−4^) and total sterol lipids (R^2^ = 0.748, FDR-adjusted *p*-value = 0.0326) displayed a more pronounced increase at the later timepoint (T3). Total non-esterified fatty acids showed a trend of decrease from T_1_ to T_2_ and increase from T_2_ to T_3_, suggesting a possible role for these lipids during the later stages of pregnancy (R^2^ = 0.204, FDR-adjusted *p*-value = 0.0742).

### 3.2. Triglyceride and Diglyceride Levels Increase and Monoglyceride Levels Decrease with the Progression of Pregnancy

There was an increase in triglyceride and diglyceride levels (Table 2) and a decrease in monoglyceride levels (Table 3) from T_1_ to T_2_ and T_2_ to T_3_. There was no change in very long-chain (VLC) monoglycerides and diglycerides, but a decreasing trend in VLC triglycerides was observed from T_1_ to T_2_ and from T_2_ to T_3_.

### 3.3. Some Free Unsaturated and Saturated Sphingosine Bases’ Levels Decrease with Progression of Pregnancy

Few free sphingosine bases’ levels were also identified and investigated. We observed a significant decrease in the levels of free sphingosine bases from T_1_ to T_2_ and from T_2_ to T_3_ including Free d16:1, Free d20:0, Free d20:1, and d16:0-1-PO4 (Table 3). The Free d18:0, Free d18:1, and Free d18:2 showed a decrease from T_1_ to T_2_ then a slight increase from T_2_ to T_3_ (Table 3).

### 3.4. Free Cholesterol Levels Increase and Very Long-Chain Cholesterol Esters’ Levels Decrease with Progression of Pregnancy

Free cholesterol levels showed a gradual increase (Table 2) during pregnancy at all three timepoints, whereas VLC cholesterol esters’ levels showed a decrease (Table 3). Cholesterol esters did not show a significant difference, but an increasing trend was observed with FDR-adjusted *p*-value = 0.089.

### 3.5. Acylcarnitines and Hydroxy Acylcarnitines Showed a Decrease in Levels with the Advancement of Pregnancy

Acylcarnitines were significantly lower (Table 2) at T_2_ compared to T_1_ and a further reduction was observed at T_3_ (R^2^ = 0.508, FDR-adjusted *p*-value = 4.20 × 10^−7^). Hydroxy acylcarnitines although present in very low levels also showed a reduction (Table 2) from T_1_ to T_2_ and T_2_ to T_3_ (R^2^ = 0.262, FDR-adjusted *p*-value = 0.000192).

### 3.6. Total Choline-Containing Lipids Increase with Progression of Pregnancy 

Total choline-containing lipid, including sphingomyelin (SM) and Phosphatidylcholine (PC), levels steadily increased during pregnancy with a high R2 value of 0.77 and FDR-adjusted *p*-value of 4.31 × 10^−6^ (Figure 1). Individually, these classes also showed an increase during pregnancy, where PC showed an R^2^ value of 0.77 and an FDR-adjusted *p*-value of 3.87 × 10^−7^ (Table 2), and SM had an R^2^ value of 0.76 and an FDR-adjusted *p*-value of 0.0007 (Table 2).

### 3.7. Lysolipid Levels Decrease with Progression of Pregnancy

Total Lyso phospholipids including lyso PC and lyso phosphatidylethanolamine (PE) levels significantly decreased (Table 3) from T_1_ to T_2_ and from T_2_ to T_3_ (FDR-adjusted *p*-value= 1.24 × 10^−10^ and 0.033, respectively). Lyso PC is a partially hydrolyzed form of PC and these results indicate a different role of these biologically active lipids compared to PC during pregnancy.

### 3.8. Levels of Important Polyunsaturated Fatty Acids Decreased from T_1_ to T_2_ and Increased from T_2_ to T_3_

One of the interesting findings was in the levels of key polyunsaturated fatty acids as they decreased from T_1_ to T_2_ while increasing from T_2_ to T_3._ The FAs that showed this trend included polyunsaturated non-esterified fatty acids (NEFAs), non-esterified (NE) Linoleic acid (18:2n6), non-esterified Arachidonic acid (20:4n6), non-esterified Alpha-linolenic acid (ALA, 18:3n3), non-esterified Eicosapentaenoic acid (EPA (20:5n3), non-esterified Docosapentaenoic acid (DPA, 22:5n3), and non-esterified DHA (22:6n3) (Table 4).

### 3.9. Effect on Ratios of Different Lipid Classes and Specific Lipids during Pregnancy

We also investigated ratios of different lipids and classes to understand the patterns of association of some lipids in comparison to other lipids (Table 5). The ratio of non-esterified Arachidonic acid with DHA, the ratio of non-esterified n6 (Arachidonic + Linoleic) to n3 (ALA + EPA + DPA + DHA), the ratio of mono-unsaturated to saturated esterified FA, the ratio of mono-unsaturated to polyunsaturated esterified FA, and the ratio of n6 (Arachidonic + Linoleic) to n3 EPA+DPA+DHA (esterified) showed an increase from T_1_ to T_2_ and T_2_ to T_3_. 

The ratio of unsaturated to saturated NEFA, the ratio of mono-unsaturated to saturated NEFA, and the ratio of polyunsaturated/saturated NEFA reduced from T_1_ to T_2_ and increased from T_2_ to T_3_. The results were different based on the esterification status and the ratio of unsaturated to saturated NEFA decreasing but the ratio of unsaturated to saturated esterified increasing.

### 3.10. Potential Lipid Signatures

**Figure 2:**
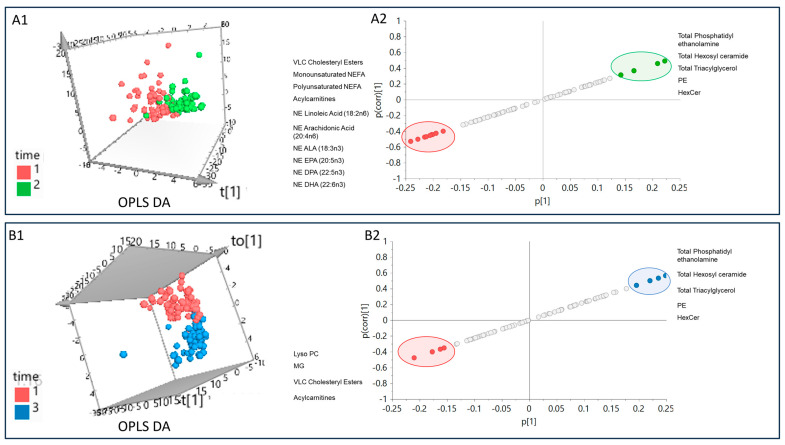
(**A**)—(**A1**) The 3D OPLS DA score plot (OPLS DA) on left showing differences in lipidome profiles at timepoints T_1_ (red) and T_2_(green). (**A2**) OPLS DA S-plot showing reliable lipid markers with high magnitude and significance; red represents higher at T_1_ and green represents higher at T_2_. (**B**)—(**B1**) The 3D OPLS DA score plot (OPLS DA) on left showing differences in lipidome profiles at timepoints T_1_ (red) and T_3_ (blue). (**B2**) OPLS DA S-plot showing reliable lipid markers with high magnitude and significance; red represents higher at T_1_ and blue represents higher at T_3_.

OPLS DA models were used to identify potential lipid signatures of pregnancy progression (Figure 2A,B). Clear groupings of T_1_ and T_2_ were observed indicating differences in lipidome profiles (R2 = 0.6, Q2 = 0.4), and an S-plot was used to identify potential lipid markers responsible for the differences in profiles of two groups (Figure 2(A1,A2)). The reliable lipid signatures identified by the S-plot (Figure 2(A2)) showed a decrease in the levels of VLC Cholesteryl Esters, monounsaturated NEFA, polyunsaturated NEFA, Acylcarnitines, NE Linoleic acid (18:2n6), NE Arachidonic acid (20:4n6), NE ALA (18:3n3), NE EPA (20:5n3), NE DPA (22:5n3), and NE DHA (22:6n3) from T_1_ to T_2_ and increase in the levels of total Phosphatidylethanolamine (which includes lyso PE, etc.), total Hexosyl ceramide, total Triacylglycerol, PE, and HexCer from T_1_ to T_2_. 

Similarly, separation and differences were observed in the lipidome profiles of T_1_ and T_3_ (R2 = 0.6, Q2 = 0.6), and the S-plot identified lipids responsible for this separation that can be used to serve as potential biomarkers (Figure 2(B1,B2)). The potential lipid markers identified by the S-plot (Figure 2(B2)) included lower levels of Lyso PC, MG, VLC Cholesteryl Esters, and Acylcarnitines from T_1_ to T_3_ and higher levels of total Phosphatidylethanolamine, total Hexosyl ceramide, total triacylglycerol, PE, and HexCer from T_1_ to T_3._

The OPLS DA model showed differences between T_2_ and T_3_ and had very low predictive value, and it was not further investigated to identify lipid signatures for differences between these two timepoints (R2 = 0.6 and Q2 < 0.1), as seen in Supplementary Figure S1.

## 4. Discussion

Lipid profiles are not regularly investigated during pregnancy. There is limited information available regarding changes in lipids across pregnancy and not many studies have investigated comprehensive lipid profiles at three timepoints during pregnancy, specifically in pregnant Black women. Mapping lipid trends could lend support to a customized health approach for high-risk pregnancies or individuals with previous high-risk pregnancies. This is particularly important for Black women who are at higher risk for adverse birth outcomes (e.g., preterm birth) [25]. In our study, we investigated the longitudinal progression of lipid levels and the relationship between lipid classes. The comprehensive investigation of lipidome profiles in the current study provided unique insight into individual lipid classes and changes in lipid metabolism with full-term pregnancy in pregnant Black women. We have found specific patterns in the lipid classes for these women. Some lipid classes increased with the progression of pregnancy; some lipid classes decreased at the later part of the pregnancy; and others showed unique patterns with levels decreasing in the middle part of the pregnancy then increasing towards the later part of the pregnancy suggesting increased requirements. We have also identified reliable lipid signatures of pregnancy progression by using multivariate dimensionality reduction models.

Total lipid values change across pregnancy and lipid requirements increase to meet fetal demand. Studies on selected lipid classes including cholesterol, triglycerides, and lipoproteins have shown increases in the levels in the latter third of pregnancy that may result from catabolism of early pregnancy adipose tissue stores that coincide with the rapid growth rate of the fetus [3]. Our analysis also demonstrates that total lipid levels increased across pregnancy. This corresponds to the existing literature that recognizes an increase in maternal adiposity and circulating lipids in the first and second trimester in order to support the growing fetus [26,27,28]. In our cohort, total lipid levels increased during pregnancy, including phospholipids. The phospholipids are a major class of lipids that include phosphatidic acid, phosphatidylcholine, phosphatidylethanolamine, phosphatidylglycerol, phosphatidylinositol, phosphatidylserine, sphingomyelin, lysophosphatidic acid, lysophosphatidylcholine, and lysophosphatidylethanolamine. Specifically, circulating phospholipids, phosphatidylcholine, phosphatidylethanolamine, phosphatidylglycerol, and phosphatidylinositol increased over time. We recently reported associations between lower phosphatidylcholine levels (measured at 9–25 weeks gestation), depressive symptoms, and preterm birth [16]. A lipidome analysis from a cohort of pregnant Puerto Rican women (24–28 weeks gestation) showed that phospholipid classes have the greatest number of metabolite-specific signatures with pregnancy outcomes such as gestational age at birth, spontaneous preterm birth, and large for gestational age neonates [12].

One of the interesting findings of the study is the change in essential and other polyunsaturated FA levels: a decrease is observed during the early to middle pregnancy and an increase is observed in the later part of the pregnancy. Fetal growth and development require essential fatty acids (EFAs; linoleic acid, omega-6, alpha-linolenic acid, and omega-3) and long-chain polyunsaturated fatty acids (LCPUFAs) derived from maternal circulation (Herrera and Ortega-Senovilla 2010 [29]). LCPUFAs are particularly important for nervous system development and are critical for brain and retinal functioning (Herrera and Ortega-Senovilla 2014 [30]). Maternal LCPUFAs (e.g., arachidonic acid; docosahexaenoic acid) are crucial for fetal development. FAs and LCPUFAs decrease in maternal circulation and increase in fetal plasma via placental transfer (Herrera and Ortega-Senovilla 2010 [29]). Arachidonic acid with DHA is required for brain and retina development and for visual activity during late pregnancy [31]. Research studies have linked maternal intake and levels of FAs to determine the availability for fetal development and neurocognitive growth [32].

Similarly, acylcarnitines are required for the proper growth of the fetus and studies have shown a reduction in the maternal levels during pregnancy due to increased fetal demand [33,34]. The decrease in acylcarnitine and hydroxy acylcarnitine levels we observed with the progression of pregnancy is consistent with the earlier reports of lower total carnitine levels during pregnancy in plasma and whole blood [33]. The researchers reported that the percentage of acylcarnitine of the total carnitine levels was equivalent to the secondary carnitine deficiency; however, they reported no change in short-chain acylcarnitine levels [33]. Acylcarnitine levels are reported to be related to maternal metabolic diseases and birth outcomes, and higher levels are reported to be associated with gestational diabetes [35,36,37]. Acylcarnitines are essential for lipid and glucose metabolism and play an important role in proper mitochondrial function [38], thus emphasizing the importance to monitor their levels during pregnancy. Our data showing reduced acylcarnitine levels with the advancement of pregnancy in pregnant Black women provides a framework to relate to pathological pregnancies in Black women.

Another class of lipids that showed a gradual increase in our cohort were sphingolipids which included ceramide and sphingomyelin. Certain species of sphingolipids like ceramides have been reported to increase toward the end of pregnancy as a signal for parturition [39]. However, elevated levels of these lipids have been linked to adverse pregnancy outcomes such as preeclampsia [39].

Glycerolipids and sterols, including triacylglycerol (TG) and free cholesterol, also increased across time in our cohort. This increase corresponds to the catabolic state of adipose tissue in the third trimester [2,27]. TG has been positively correlated with infant weight and fat mass in pregnancy with well-controlled gestational diabetes mellitus (GDM) [40]. In a population of mostly Hispanic pregnant women with spontaneous preterm birth, women who were underweight or obese pre-pregnancy showed higher diacylglycerols (DGs) and TG at 15–17 weeks of gestation [41].

Non-esterified fatty acids (NEFAs) decreased from the first timepoint to mid-pregnancy and then increased toward the end of pregnancy in our cohort. This observation correlates with the breakdown of sterol lipids toward the end of pregnancy. Maternal NEFAs may affect insulin resistance and secretion [42]. One meta-analysis found higher plasma levels of NEFAs among women with GDM compared to healthy pregnant women, specifically during the second trimester [43]. These authors proposed longitudinal measurement of NEFAs during pregnancy as a predictive tool for GDM. Aung and colleagues (2021) found that mono- and polyunsaturated NEFAs were associated with an increased risk for spontaneous preterm birth. When compared to women with obesity who delivered at term, women with obesity who experienced spontaneous preterm birth showed higher levels of NEFAs at 15–17 weeks gestation [41]. Non-esterified arachidonic acid decreased in our study from T_1_ to T_2_ and increased from T_2_ toT_3_. During normal pregnancy, increased maternal plasma NEFA and glycerol levels reflect the breakdown of adipose tissue later in pregnancy (Herrera and Ortega-Senovilla 2010 [29]).

Considering that Black women have higher rates of pregnancy complications and adverse birth outcomes compared to other races [5] and changes in lipid profiles during pregnancy can program diseases later in life for both the mother and the baby [3,44], studying changes in lipidome profiles may be an important detail in understanding maternal and fetal outcomes. In a recent study on a cohort from Singapore, researchers investigated preconception, during pregnancy, and postpartum lipidome profiles and identified lipid markers related to maternal cardiometabolic health [45]. Our study on lipid metabolism changes during full term pregnancy in Black women may help understand the role of lipid metabolism in maternal and infant outcomes and provide a much-needed platform for relating pathological pregnancies in these understudied populations. Future research should investigate the link between lipidome profiles, maternal health, and adverse pregnancy outcomes.

Strengths and limitations: The main strength of the study is the longitudinal analysis of the lipidome profiles at three timepoints in pregnant Black women. Some may view our study’s exclusive focus on Black women as limiting the generalizability of our findings. However, Black women experience the highest rates of adverse birth outcomes. Therefore, our findings can provide a path forward in understanding the etiology of adverse birth outcomes in the group at highest risk. This was a proof-of-concept study to analyze lipidome profiles at three timepoints during pregnancy. The sample size is small and needs to be followed up with large-scale studies. We used mixed-effects models, as they are well suited for small sample size studies. Maternal characteristics and other factors reported are based on self-reported surveys. As such, medical history data were not available. Additionally, as women were recruited at the prenatal clinics during pregnancy, pre-pregnancy samples were not available to establish baseline profiles. Our study is also unable to stratify pre-pregnancy overweight/obesity and other factors due to the small sample size for this project at three timepoints in full-term pregnancies. However, the models we used to investigate effects of BMI in this cohort found no significant effects (Supplementary Table S1). Further research is needed with a larger sample size to investigate the effects of weight and other factors on lipidome profiles during pregnancy.

## 5. Conclusions

Our data showed gradual changes in lipid metabolism with the progression of normal pregnancy specific to pregnant Black women. This study provides the foundation for future research to investigate perturbations in lipid metabolism related to adverse pregnancy outcomes for pregnant Black women. Our in-depth longitudinal analysis of different lipid superclasses and subclasses using a comprehensive lipidomics approach has identified major classes of lipids which showed a gradual increase throughout the pregnancy, indicating their importance and need for healthy pregnancy outcomes and normal development of the fetus. The few prior studies that included in-depth lipidome profiles were cross-sectional in contrast to our study which included three timepoints across gestation. As studies in this area grow, it may emphasize a need for health care providers to assess lipid profiles across pregnancy to reduce adverse birth outcomes.

## Figures and Tables

**Table 1:** Maternal characteristics.

Variable	Sample Total = 64
** Maternal Age**	27.17 ± 5.68
	N (%)
** Annual Household Income**	
<USD 10,000	24 (37.5)
USD 10,000–19,999	9 (14.1)
USD 20,000–29,999	15 (23.4)
USD 30,000–39,999	10 (15.6)
USD 40,000–59,999	4 (6.3)
USD 60,000–79,999	1 (1.6)
>USD 80,000	1 (1.6)
** Education**	
<High School	8 (12.5)
High School or GED	32 (50.0
Technical/Vocational	10 (15.6)
Some College	12 (18.8)
Associate Degree	2 (3.1)
Bachelor’s degree	0 (0)
Graduate Degree	0 (0)
** Work Status**	
Working	32 (50.0)
Not Working	32 (50.0)
** Marital Status**	
Married	9 (14.1)
Living with Partner	14 (21.9)
Widowed	0 (0)
Divorced	0 (0)
Separated	3 (4.7)
Never Married	37 (57.8)
Missing	1 (1.6)

**Table 2:** Lipids that showed increased levels with the progression of pregnancy.

*Lipid Classes*	R^2^	T_1_	±SE	T_2_	±SE	T_3_	±SE	*p*-Value	FDR adj
*Phosphatidylcholine (PC)*	0.769	3643.167	208.363	4225.387	208.912	4529.197	210.058	3.96 × 10^−8^	3.87 × 10^−7^
*Phosphatidylethanolamine (PE)*	0.578	262.877	18.166	380.557	18.249	453.77	18.422	6.00 × 10^−20^	5.28 × 10^−18^
*very long chain PE (VLC PE)*	0.561	2.693	0.109	3.067	0.109	3.183	0.11	1.30 × 10^−5^	6.00 × 10^−5^
*Phosphatidylglycerol (PG)*	0.388	0.504	0.046	0.612	0.047	0.682	0.047	3.26 × 10^−3^	7.00 × 10^−3^
*Phosphatidylinositol (PI)*	0.324	34.521	4.082	40.24	4.109	50.286	4.166	5.34 × 10^−3^	1.09 × 10^−2^
*Cardiolipin (CL)*	0.41	0.725	0.074	0.913	0.074	1.115	0.075	2.57 × 10^−5^	1.03 × 10^−4^
*Ceramide(Cer)*	0.56	11.774	0.653	12.445	0.656	14.288	0.662	2.90 × 10^−4^	8.80 × 10^−4^
*Hexosylceramide (HexCer)*	0.566	1.747	0.151	2.428	0.152	2.818	0.153	4.62 × 10^−11^	1.02 × 10^−9^
*Lactosylceramide (LacCer)*	0.415	2.873	0.213	2.988	0.214	3.499	0.217	2.06 × 10^−2^	3.70 × 10^−2^
*Sphingomyelin (SM)*	0.762	771.652	52.867	813.585	53.01	925.087	53.308	2.10 × 10^−4^	6.61 × 10^−4^
*Sulfatide (ST)*	0.188	0.71	0.072	0.808	0.072	1.046	0.074	1.47 × 10^−3^	3.60 × 10^−3^
*Diacylglyceride (DG)*	0.579	3.921	0.292	4.601	0.293	5.671	0.296	1.40 × 10^−8^	1.54 × 10^−7^
*Triacylglyceride (TG)*	0.648	1180.275	90.545	1473.203	90.897	1749.301	91.632	1.58 × 10^−10^	2.78 × 10^−9^
*Free Cholesterol*	0.731	372.884	22.067	384.6	22.133	423.049	22.273	7.86 × 10^−3^	1.57 × 10^−2^
*Esterified Saturated Fatty Acids*	0.473	409.917	25.249	468.361	25.388	510.028	25.677	9.80 × 10^−4^	2.54 × 10^−3^
*Esterified Mono-Unsaturated Fatty Acids*	0.502	417.729	26.27	486.409	26.408	539.557	26.696	5.87 × 10^−5^	2.07 × 10^−4^
*Esterified Poly-Unsaturated Fatty Acids*	0.784	3700.041	209.984	4191.596	210.502	4478.011	211.584	7.76 × 10^−7^	4.88 × 10^−6^
*Esterified Linoleic Acid (18:2n6)*	0.781	941.75	51.078	1012.182	51.206	1081.395	51.473	4.40 × 10^−4^	1.24 × 10^−3^
*Total Diradyl Phospholipids*	0.77	4029.496	228.964	4750.771	229.563	5146.391	230.815	4.18 × 10^−10^	6.13 × 10^−9^
*Total Choline Lipids (PC+SM)*	0.77	4514.318	259.749	5117.155	260.43	5521.277	261.851	6.37 × 10^−7^	4.31 × 10^−6^
*Total Amine Phospholipids (PE+PS)*	0.545	365.849	23.226	494.358	23.339	570.903	23.575	8.68 × 10^−15^	3.82 × 10^−13^
*Total Glycolipids (PI, HexCer, LacCer, ST)*	0.925	274.343	15.714	281.372	15.727	295.719	15.756	2.90 × 10^−3^	6.38 × 10^−3^

**Table 3:** Lipids that showed decreased levels with the progression of pregnancy.

*Lipid Classes*	R^2^	T_1_	±SE	T_2_	±SE	T_3_	±SE	*p*-Value	FDR adj
*Lyso phosphatidylcholine (Lyso PC)*	0.514	87.989	3.877	67.511	3.897	57.243	3.939	4.23 × 10^−12^	1.24 × 10^−10^
*Lyso phosphatidylethanolamine (Lyso PE)*	0.551	25.135	1.285	23.256	1.291	21.564	1.304	0.017627	0.033003
*Free d16:1*	0.506	0.524	0.058	0.254	0.058	0.247	0.058	0.00000142	0.00000835
*Free d18:0*	0.261	0.564	0.061	0.275	0.061	0.359	0.062	0.000508	0.001396
*Free d18:1*	0.218	19.84	3.213	9.582	3.236	11.14	3.285	0.026302	0.044511
*Free d18:2*	0.294	3.7	0.364	1.755	0.367	1.968	0.372	0.0000167	0.0000734
*Free d20:0*	0.486	0.182	0.025	0.116	0.025	0.091	0.025	0.001278	0.003214
*Free d20:1*	0.512	1.651	0.207	1.066	0.208	0.949	0.21	0.001812	0.004196
*d16:0-1-PO4*	0.256	0.031	0.005	0.017	0.005	0.012	0.005	0.008166	0.015969
*Monoacylglycerol (MG)*	0.148	0.155	0.015	0.063	0.016	0.043	0.016	0.000000244	0.00000195
*VLC Cholesteryl Esters*	0.56	0.167	0.017	0.08	0.017	0.062	0.017	1.06 × 10^−9^	1.34 × 10^−8^
*Acylcarnitines*	0.508	0.269	0.019	0.173	0.019	0.164	0.019	4.77 × 10^−8^	0.00000042
*Hydroxy acylcarnitines*	0.262	0.062	0.01	0.016	0.01	0.013	0.01	0.0000515	0.000192
*Total Lyso Phospholipids*	0.896	348.944	15.99	327.214	16.009	319.687	16.05	0.000341	0.001

**Table 4:** Lipids that showed a decrease during mid-pregnancy and an increase again near the term (T3).

*Lipid Classes*	R^2^	T_1_	±SE	T_2_	±SE	T_3_	±SE	*p*-Value	FDR adj
*Polyunsaturated non-esterified fatty acids (NEFA)*	0.262	96.688	9.402	66.664	9.469	93.804	9.606	0.018324	0.033594
*Non-esterified Linoleic Acid (18:2n6)*	0.249	79.991	8.169	55.893	8.227	80.024	8.348	0.025248	0.043566
*Non-esterified Arachidonic Acid (20:4n6)*	0.452	4.925	0.32	3.387	0.322	3.959	0.326	5.23 × 10^−5^	0.000192
*Non-esterified Alpha-linolenic acid (ALA) (18:3n3)*	0.189	5.717	0.609	3.48	0.613	5.179	0.623	0.013296	0.025436
*Non-esterified Eicosapentaenoic acid (EPA) (20:5n3)*	0.307	0.143	0.014	0.087	0.014	0.098	0.014	0.001595	0.003793
*Non-esterified Docosapentaenoic acid (DPA) (22:5n3)*	0.332	1.069	0.088	0.636	0.088	0.737	0.089	9.94 × 10^−5^	0.000336
*Non-esterified Docosahexaenoic acid (DHA) (22:6n3)*	0.455	2.389	0.189	1.465	0.19	1.675	0.193	1.90 × 10^−5^	7.94 × 10^−5^

**Table 5:** Changes in the ratios of lipids with the progression of pregnancy.

*Lipid Classes*	R^2^	T_1_	±SE	T_2_	±SE	T_3_	±SE	*p*-Value	FDR adj
*Ratio non-esterified Arachidonic/DHA*	0.406	2.386	0.112	2.73	0.113	2.849	0.114	0.00077	0.00205
*Ratio non-esterfied n6 Archidonic+Linoleic/n3 ALA+EPA+DPA+DHA*	0.339	9.941	0.416	12.16	0.419	12.152	0.425	3.06 × 10^−6^	1.58 × 10^−5^
*Ratio Mono-Unsaturated/Saturated Esterified FA*	0.373	1.017	0.008	1.04	0.008	1.057	0.008	0.00017	0.00055
*Ratio Mono-Unsaturated/Poly-Unsaturated Esterified FA*	0.29	0.113	0.004	0.116	0.004	0.124	0.004	0.02344	0.04126
*Ratio n6 Archidonic+Linoleic/n3 EPA+DPA+DHA (Esterified)*	0.677	2.254	0.045	2.278	0.045	2.373	0.046	0.0042	0.0088
*Ratio Arachidonic/DHA (Esterified)*	0.627	1.068	0.026	0.995	0.026	1.001	0.026	0.002439	0.0055
*Ratio Unsaturated/Saturated NEFA*	0.121	2.01	0.079	1.466	0.08	1.59	0.081	1.99 × 10^−6^	1.09 × 10^−5^
*Ratio Mono-Unsaturated/Saturated NEFA*	0.174	1.076	0.046	0.795	0.046	0.866	0.047	1.29 × 10^−5^	6.00 × 10^−5^
*Ratio Poly-Unsaturated/Saturated NEFA*	0.091	0.934	0.036	0.671	0.036	0.724	0.037	3.78 × 10^−7^	2.77 × 10^−6^

## Data Availability

Supplementary data, tables, figures, and models for this study are available at the link https://figshare.com/s/b6226d01c2519920f463, accessed on 9 March 2024 last edited 5 June 2024) Data used in this study are available (deidentified) by contacting corresponding author Nadia Saadat (saadatn@umich.edu) at reasonable request.

## Supplementary Materials

The following supporting information can be downloaded at https://figshare.com/s/b6226d01c2519920f463 (accessed on 19 March 2024 last edited 5 June 2024) Supplementary Figure S1: A—OPLS DA score plot showing less separation of the timepoint 2 and 3. One score represents one sample; B—OPLS DA score plot showing improvement of the separation between timepoint 2 and 3 after removing one outlier at both timepoint. One score represents one sample. Table S1: *p*-values Time & BMI.
